# A novel *ABCC6* variant causative of pseudoxanthoma elasticum

**DOI:** 10.1038/s41439-019-0062-x

**Published:** 2019-06-20

**Authors:** Gianluca Contrò, Rossana Tallerico, Vincenzo Dattilo, Fernanda Fabiani, Maria Vittoria Enzo, Uros Hladnik, Stefano Dastoli, Steven Paul Nisticò, Emma Colao, Nicola Perrotti, Rodolfo Iuliano

**Affiliations:** 1 Medical Genetics Unit, University “Magna Graecia”, Catanzaro, Italy; 2 Department of Health Sciences, University “Magna Graecia”, Catanzaro, Italy; 3 Medical Genetics Unit, “Mauro Baschirotto” Institute for Rare Disease, Vicenza, Italy; 4 Dermatology Unit, University “Magna Graecia”, Catanzaro, Italy

**Keywords:** Medical genetics, Diseases

## Abstract

Pseudoxanthoma elasticum is an autosomal recessive heritable disorder caused by mutations in *ABCC6*. We describe two siblings showing typical skin lesions and a clinical diagnosis of pseudoxanthoma elasticum. Genetic analysis of *ABCC6* revealed a novel homozygous c.4041G > A variant located in the last position of exon 28 that compromises the splicing donor site, resulting in a shorter messenger RNA. The deletion impairs the nucleotide-binding fold region, which is crucial for *ABCC6* function.

Pseudoxanthoma elasticum (PXE, OMIM 264800) is a rare genetic condition with skin, ocular, gastrointestinal, and cardiovascular involvement. Its prevalence is 1:25,000 to 1:100,000, and affected patients exhibit heterogeneous clinical manifestations. The first and most typical alterations are detected on the skin, where small yellowish papules appear during childhood and adolescence, slowly increasing in number and size, and usually evolving into plaques^1^. These lesions are usually located on the neck, axilla, periumbilical area, and antecubital fossa, whereas the skin above the flexural area is loose and wrinkly. Histological analysis of tissue from this area can show calcified elastic fibers and can be used as a diagnostic test revealing calcium deposition after von Kossa staining^2^. The eye is the second most common involved organ, specifically the elastic portion of Bruch’s membrane, the inner part of choroid placed just under the retina. This could cause hemorrhage and subretinal neovascularization, resulting in the loss of normal vision. Finally, the fragmentation and calcification of elastic fibers in the medium-sized arteries could cause intimal fibrosis leading to vascular occlusive disease or an increase in cardiac events. Gastrointestinal bleeding has also been reported as a sign of the illness and is included in the clinical criteria for the diagnosis of PXE^3^.

The causative gene of PXE was identified as adenosine-binding protein cassette 6 (*ABCC6*)^4^, located on chromosome 16p13.1, encoding a transporter that carries various molecules across extra- and intracellular membranes. A wide range of *ABCC6* mutations was previously reported as causative of different medical conditions characterized by altered mineralization in different tissues^5^. In this study, we detected a novel *ABCC6* variant associated with PXE in two siblings, and thus clarified the functional effect of this variant.

The first patient was an 18-year-old woman referred to our unit for the presence of the PXE typical skin alteration, at the age of 17, located on the lateral side of the neck, the axilla, and the upper side of the chest. Physical examination revealed that papules situated on the neck had formed a plaque with a diameter of 8 cm; the few other papules on the axilla and chest were isolated (Fig. 1a). Fundus ocular examination showed the presence of asymptomatic angioid streaks. No other signs were detected in either the ocular or the cardiovascular areas after examination with ECG and ECO.

**Fig. 1:**
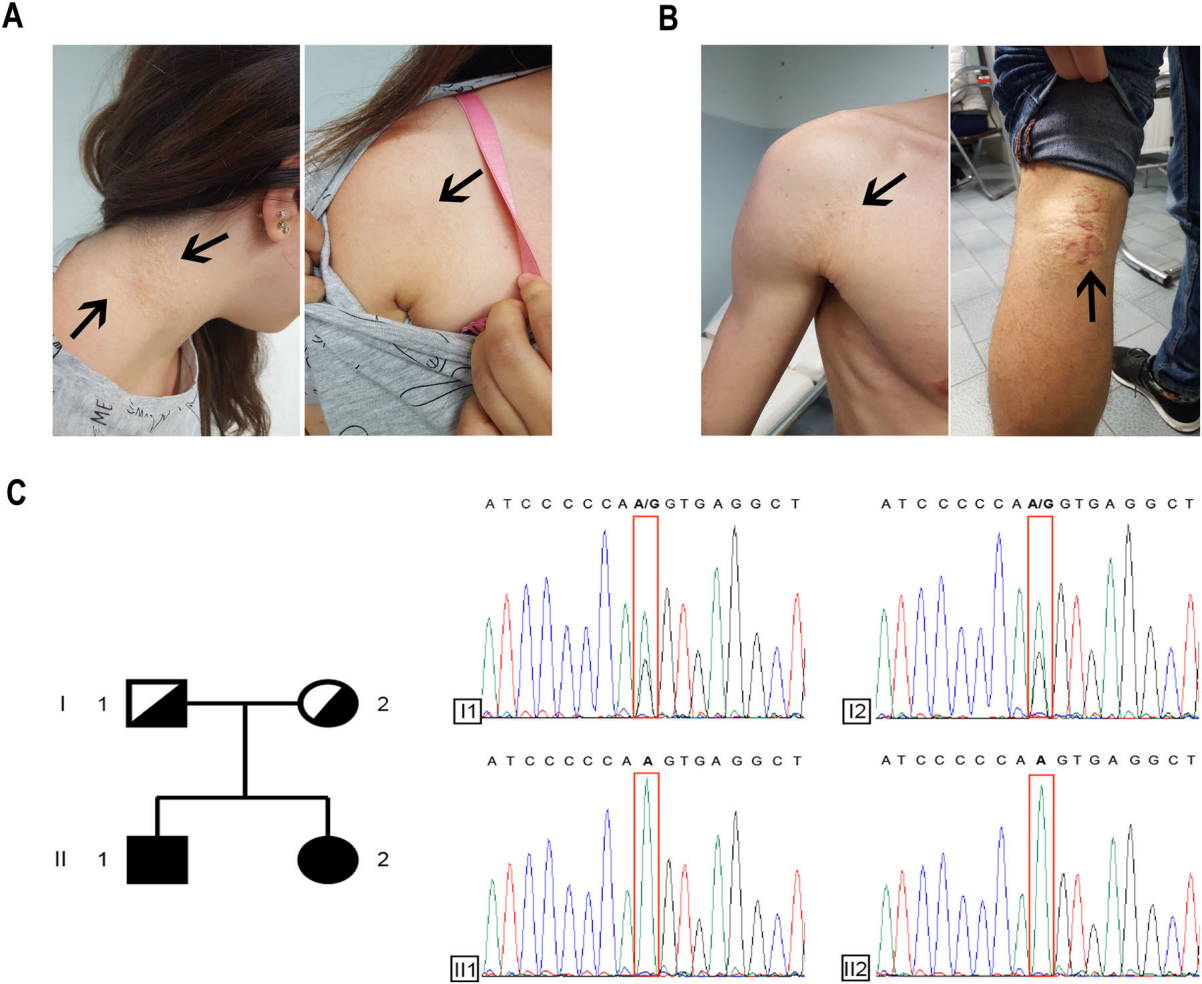
**a** Papules on the neck and axilla of patient 1; **b** lesions on the shoulder and popliteal fossa of patient 2. **c** Pedigree of the family with electropherograms showing the DNA sequence surrounding the site of the c.4041 G > A variant (I1, father; I2, mother, II1, patient 2; and II2, patient 1). The variant is highlighted by a red rectangle

The second patient was 23 years old, the brother of patient 1. He showed, at the time of his visit, only small yellowish papules on the shoulder and at the popliteal fossa (Fig. 1b). The patient indicated that the onset of these papules was 1 year prior. No other sign was detected at the physical examination, including ocular or cardiovascular signs after the examination of the ocular fundus or after ECG and ECO. The papules localized on the popliteal fossa were infected due to the friction above the area. Informed written consent was obtained from all the participants in this study. The institutional review board approved the study protocol.

Genomic DNA from patient 1 was extracted from blood sample with a commercial DNA extraction kit (Nuclear Laser Medicine, Milano, Italy) and analyzed by Sanger sequencing of all 31 exons with the flanking intronic sequences of *ABCC6* (NM_001171.5). A quantitative evaluation of the area of the *ABCC6* was performed by multiplex ligation-dependent probe amplification (MLPA). The identified variant was then investigated in patient 2 (her sibling) and their parents by targeted Sanger sequencing. High-specificity primer sets were used for the PCR amplification of all exons of *ABCC6* to avoid co-amplification of the high-homology pseudogenes. PCR products were bidirectionally sequenced with the ABI BigDye Terminator Cycle Sequencing Kit V3.1 on a 3130 ABI PRISM Genetic Analyzer (Applied Biosystems, USA). MLPA was performed using the SALSA MLPA P092 *ABCC6* probemix (MRC Holland, Amsterdam, the Netherlands).

Peripheral blood mononuclear cells (PBMCs) were isolated via density gradient centrifugation in Biocoll Separating Solution (Biochrom GmbH, Berlin, Germany). RNA extraction from PBMCs was performed using the RNeasy Mini Kit (Qiagen, Valencia, CA, USA) following the manufacturer’s instructions. One microgram of total RNA from each sample was subjected to reverse transcription using the High Capacity RNA-to-cDNA Kit (Applied Biosystems, Foster City, CA, USA) according to the manufacturer’s instructions. Specific primers 5′-TTCAAGATCCACGCAGGAGA-3′ (forward in exon 27 of *ABCC6*) and 5′-CAGTGCACTGTGCAAACCA-3′ (reverse in exon 30 of *ABCC6*) were used to amplify cDNA by PCR. The PCR product obtained was visualized on a 1.5% agarose gel and then purified, and subjected to bidirectional sequencing.

Provean software^6^ and Human Splicing Software web source version 3.1^7^ were used to predict the possible effect of the variant detected.

The amino acid sequence of adenosine-binding protein cassette 6 protein was obtained from the UniProt database^8^. Homology modeling techniques were utilized to construct the three-dimensional (3D) model of *ABCC6* from the atomic coordinates of Protein Data Bank entry 6BZS using the UCSF Chimera Program^9^.

MLPA showed no alteration in the exon copy number of *ABCC6* in patient 1.

Analysis of all *ABCC6* exons with flanking intronic sequences detected, in patient 1, a novel c.4041 G > A variant, located in exon 28, and in an apparent homozygous state (Fig. 1c). The variant has been submitted to the ClinVar database with the accession code SCV000864171. MLPA analysis showed no copy number variations of *ABCC6* exons in patient 1, confirming the homozygous state of the variant. Sequencing of the *ABCC6* exon 28 in patient 2 revealed the same homozygous variant. To establish the genotype of their parents, they were recruited for DNA sequencing of the *ABCC6* exon 28. As expected, both parents were heterozygous healthy carriers of the c.4041 G > A variant (Fig. 1c). This variant has a frequency of 2/241074 alleles in the combined gnomAD database (Genome Aggregated Database, Broad institute, Cambridge, MA, USA)^10^, suggesting that it is not a simple polymorphism. The variant does not lead to an amino acid change, but it is located in the last position of exon 28 of *ABCC6*, potentially affecting the donor splice site. Thus, we used Human Splicing Finder software to assess whether the variant could alter the splicing process, and we found that the splice-potential score of the donor splice site decreases from 91.22 to 62.27 (−31.74%) in the mutated sequence. This result prompted us to perform RT-PCR which revealed a shorter *ABCC6* messenger RNA in the affected patient compared with two healthy controls (Fig. 2a). The sequencing of the PCR product demonstrated that, in patient 1, the whole exon 28 of *ABCC6* messenger RNA is skipped (Fig. 2b), generating an in-frame protein lacking 53 amino acids at positions 1295–1347 (p.Val1295_Gln1347del). This deletion is predicted to be deleterious, with an elevated score (−214.379; threshold value of −2.5) by the Provean software. To investigate the possible effect of the p.Val1295_Gln1347del variant, 3D modeling was carried out to examine putative structural changes caused by this variant. The resulting mutated protein impairs nucleotide-binding fold 2 (NBF2) domain structure compared to the wild-type form (Fig. 2c).

**Fig. 2:**
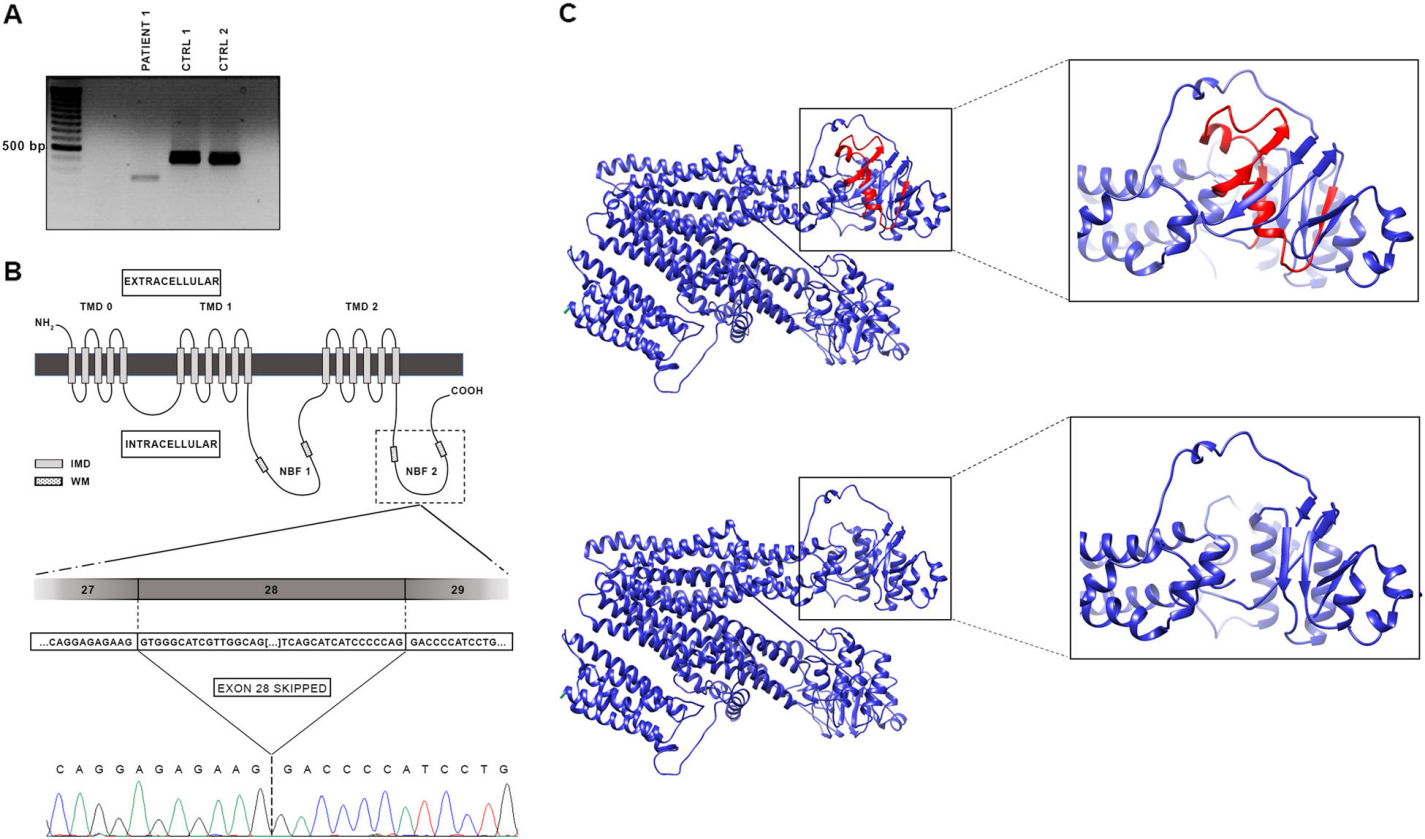
**a** Agarose gel electrophoresis of RT-PCR, performed with primers complementary to sequences in exons 27 (forward primer) and 30 (reverse primer), showing a shorter amplified product in Patient 1 compared with those obtained in two healthy controls. **b** *ABCC6* consists of three transmembrane domains (TMD0, TMD1, and TMD2) and three intercellular loops; two of these are the nucleotide-binding folder (NBF), characterized by the presence of two Walker motifs (WM). Exon 28 is a part of NBF2 (dotted square). Comparison of the *ABCC6* wild-type sequence with the mutated sequence. (IMD = intermembrane domain). **c** Predicted three-dimensional structure of the *ABCC6* protein. The upper panel shows the wild-type model and the magnification of the deleted amino acid sequence (highlighted in red). The predicted structure of the mutated protein and the relative magnification, lacking the deleted part, are shown in the lower panel

*ABCC6* encodes a 165 kDa protein of 1503 amino acids and is a putative efflux cellular transporter^11^. The structure of the protein consists of 17 transmembrane domains and two intracellular NBF, which are critical regions for ATP binding and hydrolysis^12^. In *ABCC6*, the binding domains are encoded by exons 17–18 and exons 28–29–30. Thus, exon 28, which is skipped in *ABCC6* messenger RNA because of the novel variant described in this paper, belongs to a protein region containing the ATP-binding domain. This suggests that, in the mutant form, the transport function and the ATPase activity are impaired, leading to the development of the disease. A former identification of a mutation at the same site supports our hypothesis^13^. The previously described variant is a substitution of a guanine into a cytosine at position 4041, which theoretically leads to a substitution of a glutamine with a histidine at position 1347. Considering the alteration of the splice site, the exclusion of exon 28 during splicing is also a plausible hypothesis that was reported but not demonstrated. The critical role of exon 28 of *ABCC6* was demonstrated “in vitro” by the detection of a significant decrease in the *ABCC6* transport activity occurring in three mutants generated with changes in residues encoded by the exon 28^14^. In the European population, two mutations are reported as the most recurring. The first one is p.Arg1141X in exon 24 with a prevalence of 30% of all PXE mutations; the second one is a deletion of exons 23–29, found in 20% of the patients with PXE^15,16^. In both cases, the second NBF of *ABCC6* is lacking. The identified variant further underlines the importance of the second NBF in the normal activity of *ABCC6*. It also confirms once again that the analysis of the *ABCC6* should include all the coding exons and should not be limited to a selected few hotspot regions.

## Data Availability

The relevant data from this Data Report are hosted at the Human Genome Variation Database at 10.6084/m9.figshare.hgv.2582.
